# The problem of unconscious and unidentified patients in emergency department admissions; a 3-year retrospective study

**DOI:** 10.1371/journal.pone.0307540

**Published:** 2024-07-24

**Authors:** Demet Acar, Fatih Cemal Tekin

**Affiliations:** Department of Emergency Medicine, Konya City Hospital, Karatay, Konya, Türkiye; University of Health Sciences, Beyhekim Training and Research Hospital, TÜRKIYE

## Abstract

Unidentified patients present a medical information dilemma for all medical departments but can be a major problem in Emergency Departments (EDs). This study aimed to determine the clinical and socio-demographic profile of ’unidentified’ patients admitted to the ED with altered consciousness and to define the outcomes of these patients. All ED presentations were analyzed retrospectively for the unidentified patients brought to the hospital by ambulance with altered consciousness. We assessed demographic data, clinical presentation, discharge information, and major clinical outcomes. In this study, 1324 unidentified patients were admitted with altered consciousness to the ED. Of these, 1048 (80.1%) were foreign nationals. In this patient group, the most common diagnoses were; traffic accidents, assault or sharp object injuries, drug addicts, or syncope-epilepsy. In addition, the number of patients who left the hospital without permission or escaped and therefore could not be diagnosed was higher in the foreign nationalities group and constituted approximately one-fifth of the patients (18.9% vs. 5.4%, p:0.001). Of the unidentified patients, 903 (68.2%) were discharged after treatment. 351 (26.5%) patients left the ED unattended. 32 (2.4%) patients were hospitalized. 38 (2.9%) patients died in ED. The majority of the unidentified patients admitted to the ED with altered consciousness were immigrant males. Unidentified patients are a high-need population, most commonly presenting with substance misuse or trauma. Although most of the patients were seeking urgent treatment, more than one-fourth of the patients left the hospital without appropriate treatment and most of these patients were also immigrants. We believe that economic, linguistic, and social disadvantages played an important role in this outcome.

## Introduction

Emergency departments (EDs) are intended to provide fast, convenient, high-quality, and unscheduled care for urgent cases [1]. Altered consciousness is a state of reduced alertness or inability to arouse due to low environmental awareness, which is an important reason for ED admission. Trauma, neurological disease, metabolic disease, or intoxications are the main causes of altered consciousness in the ED. Since presentations to the emergency department with these pathologies are generally acute and unprepared, emergency physicians frequently encounter anonymous cases. The accompanying alterations in the patient’s consciousness also complicate the collection of medical histories. Therefore, understanding and predicting the demographic characteristics of such patients becomes even more crucial [2,3].

The unidentified patients present a medical information dilemma for all medical departments but can be a major problem in emergency departments, as these patients often require urgent diagnosis and intervention in the absence of medical records. In a previous study, unidentified patients were reported to be the most severely ill, with significantly more admissions to skilled nursing facilities and long-term care hospitals, as well as increased respiratory morbidity and longer hospital stays, compared with matched controls [4,5]. There may also be confusion among health professionals, which may lead to poor care when caring for injured, unidentified patients, which may also prolong the time it takes to provide the most appropriate treatment [5]. For all these reasons, unidentified patients can be a major problem in emergency departments. However, data on unidentified patients admitted to EDs are very limited.

The objective of this study was to determine the clinical and socio-demographic profile of “unidentified” patients admitted to the emergency department with altered consciousness and to define the outcomes of these patients.

## Materials and methods

This retrospective study was conducted in the Konya Adult Emergency Department. After obtaining the necessary permissions, we accessed patient records and data from February 1, 2024, to April 1, 2024. We included patients who were brought to the hospital by ambulance with loss of consciousness or altered consciousness between January 1, 2021, and January 1, 2024, and whose identities could not be determined. We evaluated the demographic characteristics, clinical information, and discharge details of these patients based on the obtained records and data.

All statistical analyses were carried out using SPSS Statistics 21. Descriptive statistics were reported for all variables collected. Study data were tested for normality of distribution using the Kolmogorov-Smirnov and assessed using descriptive statistical methods (presented as mean, standard deviation, median, and interquartile range and frequency). Non-normally distributed numerical data were analyzed using the Mann-Whitney U test, whereas normally distributed data were analyzed using the Student’s t-test. Nominal data were compared using the chi-squared test. Statistical significance was set at p<0.05.

The local ethics committee approved the study, which waived the obligation to obtain informed consent (date:02/01/2024, number:03–34).

## Results

In this study period, approximately 1 million 800 thousand patients were admitted to the ED. It was found that approximately 73,000 patients were admitted with altered consciousness, of which 1324 unidentified patients were admitted to the ED during these 3 years. Of these, 1048 (80.1%) were foreign nationals. The demographic features of those unidentified patients are summarized in Table 1.

**Table 1:** Demographic features of unidentified patients admitted to the ED.

		Foreign Nationals n (%)	Turkish Citizens n (%)	p
Gender	Male	734 (70,04)	207 (75)	0.76
	Female	314 (29,96)	69 (25)	
		**mean±sd**	**mean±sd**	**p**
Age		28.12±15.07	25.17± 15.12	0.68

In this patient group, the most common diagnoses were; traffic accidents, assault or sharp object injuries, drug addicts, or syncope-epilepsy. The diagnoses of the study patients are summarized in Table 2. Assault or sharp object injuries were more common in foreign nationalities; while traffic accidents, drug addicts, and dementia are more common in Turkish citizens. In addition, the number of patients who left the hospital without permission or escaped and therefore could not be diagnosed was higher in the foreign nationalities group and constituted approximately one-fifth of the patients (18.9% vs. 5.4%, p:0.001).

**Table 2:** Diagnoses of unidentified patients in ED.

	Foreign Nationals (n:1048)	Turkish Citizens (n: 276)	p
**Traffic accidents (n, %) **	291 (27.7)	95 (34.4)	0.42
**Assault or sharp object injuries (n, %) **	312 (29.7)	38 (13.7)	**0.01**
**Drug or alcohol addicts (n, %) **	90 (8.6)	56 (20.2)	**0.01**
**Dementia (n, %) **	8 (0.76)	11 (3.9)	**0.01**
**Syncope-epilepsy (n, %) **	68 (6.5)	13(4.7)	0.63
**Carbon monoxide poisoning (n, %) **	39 (3.7)	11 (3.9)	0.82
**Suicide (n, %) **	21 (2.0)	10 (3.6)	0.74
**Natural disasters (earthquake) (n, %) **	8(0.76)	2 (0.72)	0.92
**Arrest–exitus (n, %) **	13 (1.2)	25 (9.1)	**0.01**
**Unknown—(n, %) **	198 (18.9)	15 (5.4)	**0.01**

Of the unidentified patients, 903 (68.2%) were discharged after treatment. 351 (26.5%) patients left the emergency department unattended. 32 (2.4%) patients were hospitalized. 38 (2.9%) patients died in ED. As the identity of 32 of the 38 patients who died could not be established, they were buried in the cemetery for the homeless.

## Discussion

Upon examining the demographics of unidentified patients brought to the emergency department with altered or loss of consciousness, it was noted that, in addition to trauma, substance abuse, and acute neurological diseases, the most prominent demographic feature in this patient group was the immigrant population. Approximately 70% of the patients were male, the mortality rate was 2.9%, and the ratio of patients who left the hospital without permission was as high as 26.5%. To the best of our knowledge, this is the first study in the literature evaluating unidentified patients admitted to the ED with altered consciousness.

Unfortunately, there is very little data in the literature to date on the general characteristics of unidentified patients admitted to the ED. Tastad et al [6] conducted a retrospective study in Canada and reported that unidentified patients were more likely to be male. The most common presenting complaints were substance abuse and trauma, as was the case in our study. There was a 13.2% 30-day mortality rate for all patients. Since we only recorded the outcomes in ED, we can not discuss the 30-day mortality rates in our study group. In another retrospective study, a total of 151 unidentified patients were admitted to the neurological emergency department over 10 years. Among these patients, there was also a male predominance. The most common causes were seizures, metabolic causes, and neuro-infections, and the mortality rate in this study was 9.3% [7].

**Fig 1:**
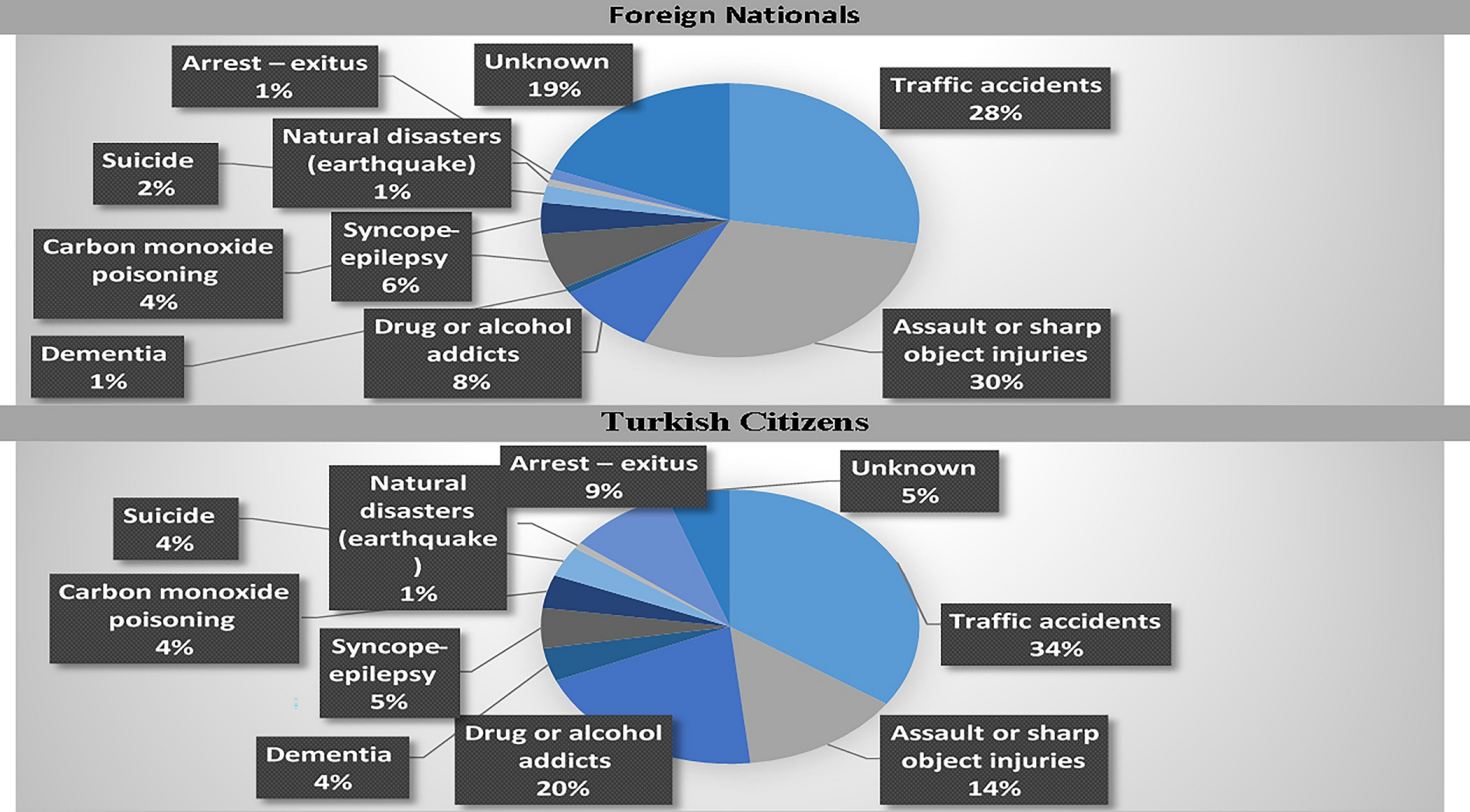
Distribution of diagnoses of unidentified patients in the emergency department.

In our study, trauma or drug intoxication was the most common cause of admission to the ED among unidentified patients (Fig 1). This is not a surprising finding, as studies of EDs of ’identified’ patients, particularly in younger age groups such as ours, have reported trauma and intoxication as the main reasons [3,8]. However, despite the majority of these patients requiring urgent care, the proportion of patients who left the hospital without permission was as high as 26.5%. This means that more than a quarter of patients left A&E without appropriate treatment and/or follow-up. This is an interesting finding, but we could not find any data on this in the previous literature.

One of the most important and striking results of this study was that a large proportion (79.2%) of the unidentified patients presenting to the ED with altered consciousness were immigrants. Unfortunately, since the region where we worked is not a very touristic region and health tourism is not very active in the emergency department, most of the foreign nationalities patients admitted to the emergency department were immigrants. In recent decades, the world has seen a dramatic increase in all types of migration which has increased linguistic and cultural diversity in many countries [9]. Our country also has a key role to play in this regard due to its geopolitical location [10,11]. The data regarding the immigrants in ED is increasing day by day. In a recent study from our country, in a year, a total of 3865 migrants were admitted to the ED, with a median age of 22 years and a male predominance. The most common diagnoses were diseases of the musculoskeletal system and connective tissue and diseases of the respiratory system [12]. A recent review reported that migrants’ access to care is hampered by language barriers, poor insurance coverage, and a lack of knowledge of the local health system [13]. In our study, the number of patients leaving the hospital without a diagnosis or permission was higher among immigrants than among Turkish citizens, and we believe that these economic, linguistic, and social disadvantages played an important role in this outcome.

The rate of patients who died in the ED in this study was 2.9%; which was not higher than the previous data about identified patients admitted to the ED with altered consciousness. In a recent study, Kim et al [14] reported that among 2028 patients admitted to the ED with altered levels of consciousness over two years, the overall mortality rate was 17.2% and 6.1% of these patients died in the ED and the remainder died on the ward or in the intensive care unit. However, this was an older group of patients with a mean age of 68.33 ± 16.31 years and this may be the reason for the higher mortality rates compared to our results. In another recent study, Jessica et al [15] reported the 7-day mortality of adult patients presenting to the ED with altered mental status as 3.2%.

This study has some limitations that should be mentioned. First, this is a single-center retrospective study, which may lead to selection bias. Second, because the patients studied in this study were unidentified, some patient records may be missing.

In conclusion, the majority of the unidentified patients admitted to the ED with altered consciousness were immigrant males. Unidentified patients are a high-need population, most commonly presenting with substance misuse or trauma. We believe that economic, linguistic, and social disadvantages played an important role in this outcome. The profile of patients that emergency physicians are likely to encounter can be determined by knowledge of the socio-demographic characteristics of unidentified patients attending the ED and information about their diagnoses. There is a need for further research into the emergency department visits of unidentified patients.

## References

[1] Alnasser S, Alharbi M, AAlibrahim A, Aal Ibrahim A, Kentab O, Alassaf W et al. Analysis of Emergency Department Use by Non-Urgent Patients and Their Visit Characteristics at an Academic Center. Int J Gen Med. 2023; 20(16): 221–232. doi: 10.2147/IJGM.S391126 36711428 PMC9880025

[2] Reichhart MD, Meuli R. Alterations of the level of consciousness related to stroke. In: Godefroy O, editor. The Behavioral and Cognitive Neurology of Stroke. Cambridge University Press; 2013. pp. 312.

[3] Cooksley T, Rose S, Holland M. A systematic approach to the unconscious patient. Clin Med. 2018; 18(1): 88. doi: 10.7861/clinmedicine.18-1-88 29436445 PMC6330912

[4] Janowak CF, Dolejs SC, Zarzaur BL. Who is John Doe? A case-match analysis. Am Surg.2017; 83(8): 294–296. 28822365

[5] Janowak CF, Agarwal SK, Zarzaur BL. What’s in a Name? Provider Perception of Injured John Doe Patients. J Surg Res. 2019; 238: 218–223. doi: 10.1016/j.jss.2019.01.027 30772680

[6] Tastad K, Koh J, Goodridge D, Stempien J, Oyedokun T. Unidentified patients in the emergency department: a historical cohort study. CJEM. 2021; 23(6): 772–777. doi: 10.1007/s43678-021-00165-0 34403119

[7] Umesh A, Gowda GS, Kumar CN, Srinivas D, Dawn BR, Botta R et al. Unknown Patients and Neurology Casualty Services in an Indian Metropolitan City: A Decades Experience. Ann Indian Acad Neurol. 2017; 20(2): 109–115. doi: 10.4103/0972-2327.205764 28615894 PMC5470161

[8] Völk S, Koedel U, Pfister HW, Schwankhart R. Impaired consciousness in the emergency department. Eur Neurol. 2018; 80(3–4):179–186. doi: 10.1159/000495363 30541008

[9] Doust Mohammadi MM, Salmani I, Farahmandnia H. Social vulnerabilities among immigrants and refugees in emergencies and disasters: a systematic review. Front Public Health. 2024; 11:1235464. doi: 10.3389/fpubh.2023.1235464 38516566 PMC10956690

[10] Pavli A, Maltezou H. Health problems of newly arrived migrants and refugees in Europe. J Travel Med. 2017; 24(4): 1–8. doi: 10.1093/jtm/tax016 28426115

[11] Gulacti U, Lok U, Polat H. Emergency department visits of Syrian refugees and the cost of their healthcare. Pathog Glob Health. 2017; 111(5): 219–224. doi: 10.1080/20477724.2017.1349061 28720037 PMC5560198

[12] Bildik B, Aker M. Demographic and Clinical Characteristics of Migrant Patients Visiting the Emergency Department. Cureus. 2023; 15(5): e39746. doi: 10.7759/cureus.39746 37398801 PMC10310547

[13] Acquadro-Pacera G, Valente M, Facci G, Molla Kiros B, Della Corte F, Barone-Adesi F et al. Exploring differences in the utilization of the emergency department between migrant and non-migrant populations: a systematic review. BMC Public Health. 2024; 24(1): 1–16. doi: 10.1186/s12889-024-18472-3 38580984 PMC10996100

[14] Kim KT, Jeon JC, Jung CG, Park JA, Seo JG, Kwon DH. Etiologies of altered level of consciousness in the emergency room. Sci Rep. 2022; 12(1): 4972. doi: 10.1038/s41598-022-09110-2 35322140 PMC8942995

[15] Stanich JA, Oliveira J E Silva L, Ginsburg AD, Mullan AF, Jeffery MM, Bellolio F. Increased short-term mortality among patients presenting with altered mental status to the emergency department: A cohort study. Am J Emerg Med. 2022; 51: 290–295. doi: 10.1016/j.ajem.2021.10.034 34785485 PMC9376886

